# Another Medical Reformer

**Published:** 1907-06-29

**Authors:** 


### Another Medical Reformer.

The indignant denouncer of medical wickedness in high places is a well-recognised figure in the ranks of the comminatory prophets. Oppressed by the persecution of his brethren, he forsakes the professional forum, and pours his story of woe and wrong into the ear of a startled and sympathetic public. For himself he has no end to serve and no axe to grind. But his heart and his adjectives are aglow with a passion for truth and justice, and no sense of self-interest, and no truckling motive, will stay the eloquence of his honest and fearless pen. Yet, explain it how one will, he comes ever to the same fate. For some brief period he lives in the glory of headlines, and then is promptly withdrawn to make way for some still newer sensation.

The latest addition to this anxious and agitated band is Dr. Forbes Ross. As has been the experience of so many of his fellow prophets, Dr. Ross has suffered from the intrigues of unscrupulous conspirators. What he now proclaims *urbi et orbi*, he had desired to discuss in the seclusion of a strictly professional audience. But his truly excellent intentions were wickedly frustrated. "Interested persons," as he darkly and gloomily describes them, intervened, and "side-tracked" his attempt to gain a hearing. What therefore remains but to appeal to public opinion and the Press? This inner history of the subterranean movements which have preceded Dr. Ross's fiery eruption we gather from a printed letter which appears to have been widely distributed to the lay press. It is a somewhat rambling and decidedly extravagant document, and many of its phrases are more vigorous than elegant. In it, several professional practices are subjected to violent, but hardly to reasonable, comment. The special discovery, however, which has moved Dr. Ross to the practice of public castigation is the recognition of a dark and dangerous combination which he names the "hospital ring." There seems to be no limit to the pernicious influence which, in its discoverer's imagination, this organisation is capable of exercising. Regarding its composition, we get little information, but the range of its activities is truly extraordinary. Though, as we are glad to learn, it does not include the "leaders of the profession," yet these so far feel its sway that personal interests and policy compel them (in a split infinitive) "to tacitly acquiesce." And the lay committees of hospitals, it seems, have been altogether mastered. They have been reduced, we are told, to the position of "powerless tools," and are "helplessly under the heel" of a corrupt section of the profession. At elections to the staffs of hospitals the secret junta commands the entire situation, and "deliberate wickedness is silently perpetrated," and "private hatreds are repaid." These are some of the directions in which Dr. Ross recognises the baleful influence of "the ring." And the object and motive of all these proceedings is, he tells us, that the members of this terrible conspiracy may drive away capable and energetic workers, and may maintain their own private practices at high fees, and safe from the influence of effective competition.

There is much more of the same sort of stuff, but the exhibition is not an attractive one. Running amok is a comparatively easy undertaking, and it seems an occupation for which Dr. Ross possesses many qualifications. Of course he will have his reward. Though we have not afforded him a full opportunity of showing his paces, we have given him a larger hospitality than his contribution merits. His one serious proposition is that no medical man shall hold more than one hospital appointment. That at least is worth discussing. But we propose to postpone its discussion until we find it associated with the ordinary decencies and courtesies of civilised life. The manner in which a cause is advocated always counts for something. Introduced in the fashion of the letter here considered the most reasonable of claims would deserve pity. In one thing Dr. Ross has certainly shown good judgment. His corybantic ravings may be appreciated in certain lay columns. But here we have for them neither room nor audience.

